# The Helminth-Derived Immunomodulator AvCystatin Reduces Virus Enhanced Inflammation by Induction of Regulatory IL-10^+^ T Cells

**DOI:** 10.1371/journal.pone.0161885

**Published:** 2016-08-25

**Authors:** Martijn J. Schuijs, Susanne Hartmann, Murray E. Selkirk, Luke B. Roberts, Peter J. M. Openshaw, Corinna Schnoeller

**Affiliations:** 1 Respiratory Science Division, National Heart and Lung Institute, Imperial College London, London, United Kingdom; 2 Department of Life Sciences, Imperial College London, London, United Kingdom; 3 Centre for Infection Medicine, Institute for Immunology, Freie Universität Berlin, Berlin, Germany; Mayo Clinic Minnesota, UNITED STATES

## Abstract

Respiratory Syncytial Virus (RSV) is a major pathogen causing low respiratory tract disease (bronchiolitis), primarily in infants. Helminthic infections may alter host immune responses to both helminths and to unrelated immune triggers. For example, we have previously shown that filarial cystatin (AvCystatin/Av17) ameliorates allergic airway inflammation. However, helminthic immunomodulators have so far not been tested in virus-induced disease. We now report that AvCystatin prevents Th2-based immunopathology in vaccine-enhanced RSV lung inflammation, a murine model for bronchiolitis. AvCystatin ablated eosinophil influx, reducing both weight loss and neutrophil recruitment without impairing anti-viral immune responses. AvCystatin also protected mice from excessive inflammation following primary RSV infection, significantly reducing neutrophil influx and cytokine production in the airways. Interestingly, we found that AvCystatin induced an influx of CD4^+^ FoxP3^+^ interleukin-10-producing T cells in the airway and lungs, correlating with immunoprotection, and the corresponding cells could also be induced by adoptive transfer of AvCystatin-primed F4/80^+^ macrophages. Thus, AvCystatin ameliorates enhanced RSV pathology without increasing susceptibility to, or persistence of, viral infection and warrants further investigation as a possible therapy for virus-induced airway disease.

## Introduction

Respiratory Syncytial virus (RSV) is the commonest single cause of hospitalization during infancy, resulting in approximately 160,000 deaths annually worldwide [1]. Infection with RSV causes inflammatory cell recruitment to the lung leading to bronchiolar occlusion [2] and RSV bronchiolitis may be associated with recurrent wheezing and asthma in later life [3,4]. Despite the great need for a vaccine, none is yet available. Vaccination with alum-adjuvanted formalin-inactivated RSV vaccine caused increased disease severity and deaths, probably associated with eosinophilic disease [5,6]. Prophylactic administration of the humanised anti-RSV monoclonal palivizumab (Synagis^®^) prevents infection in high-risk infants [7] and reduces the frequency of subsequent wheezing [8], but is costly. The great burden of RSV disease and the lack of an effective treatment underscores the importance of developing new intervention strategies.

Epidemiological studies suggest that chronic helminthic infection might protect humans from allergic sensitization and reduce allergic and inflammatory responses [9,10]. In animal models, helminthic infections prevent or cure inflammation of mucosal tissues such as lung and gut [11–14]. However, the mechanisms underlying these effects remain largely obscure and varies depending on species and disease [15]. Moreover, studies on co-infection of viruses and helminths have indicated a general impairment of antiviral immunity leading to increased virus persistence [16,17], but very little is known on the effect of specific, recombinant parasite immunomodulators in viral disease.

We have previously shown that AvCystatin, a recombinant cysteine protease inhibitor derived from the filarial nematode *Acanthocheilonema viteae*, abrogates Th2-related inflammatory disease in *in vivo* models of OVA-induced allergic airway inflammation, Th1-related inflammation in dextran sulphate sodium-induced colitis [18] and grass pollen-specific allergic responses [19]. The anti-inflammatory activity of AvCystatin appears to be mediated by enhancement of IL-10 production by host macrophages since protection is lost after macrophage depletion or blocking of IL-10 signalling [20]. In addition, the increased number of regulatory T cells (Tregs) observed in the peribronchial lymph nodes (PBLN) in the OVA allergy model suggests a possible role for Tregs in cystatin-induced suppression of inflammatory responses[18] as may be the case in other situations [11,21,22].

To test the ability of AvCystatin to modify virus-enhanced immunopathology we investigated its effects in primary RSV infection and two models of virus-induced lung eosinophilia in mice: RSV challenge of mice sensitised with recombinant vaccinia encoding the major surface glycoprotein G (vvG/RSV) [23] and RSV challenge of formalin-RSV vaccinated mice (FI-RSV/RSV) [24]. AvCystatin suppressed eosinophilic, as well as primary neutrophillic immunopathology without impairing immune defence against viral infection without altering viral clearance or virus load. Immunoprotection in either eosinophilic model correlated with induction of IL-10 producing CD4^+^ T cells; adoptive transfer of AvCystatin treated macrophages activated IL-10-secretion by CD4^+^ T cells. AvCystatin therefore selectively blocks viral immunopathology without dampening anti-viral immunity, and might be used to counter virus-enhanced immunopathology at mucosal sites.

## Results

### AvCystatin treatment reduced RSV-induced immunopathology in Th2-based viral lung eosinophilia

**Fig 1:**
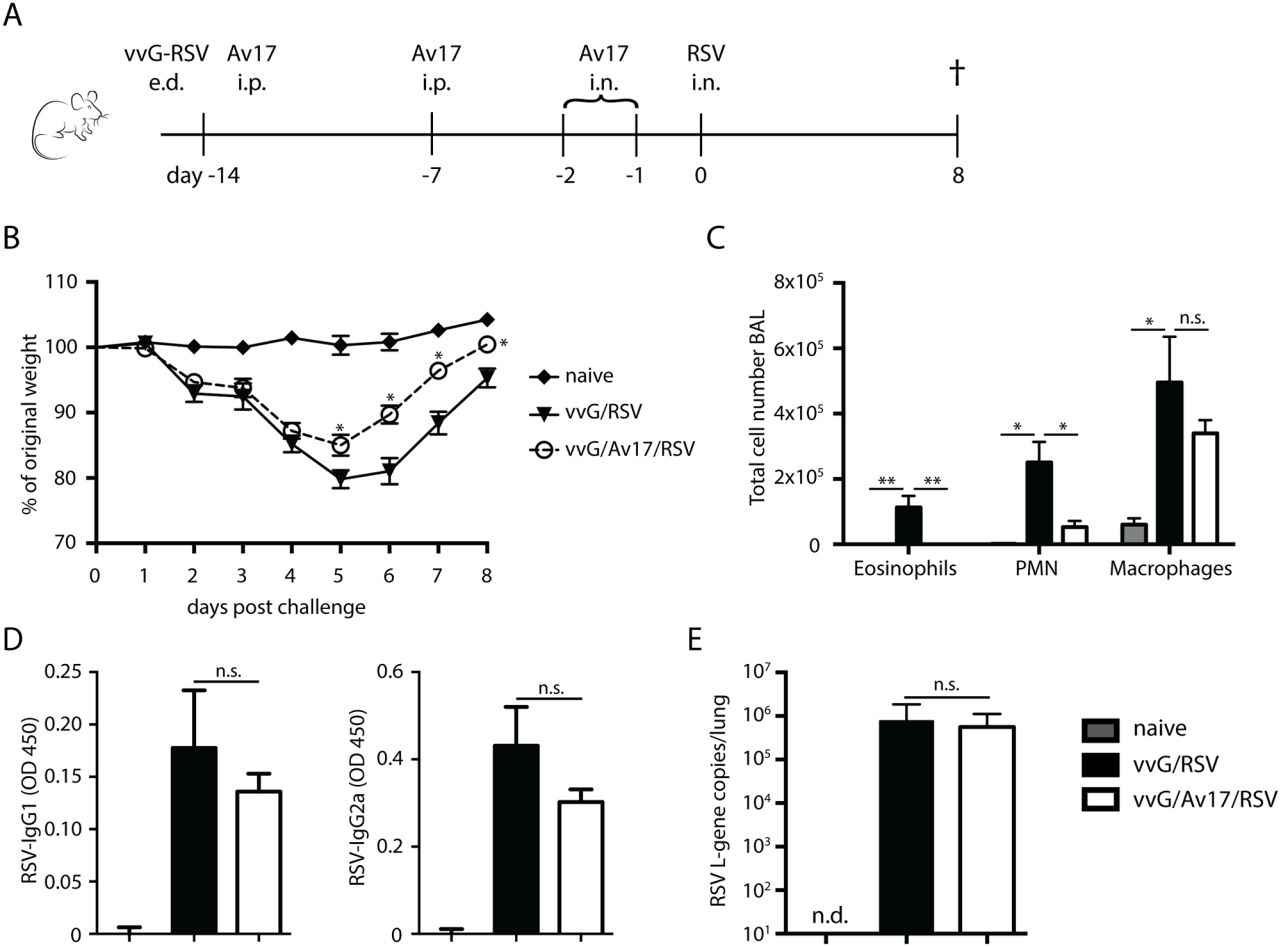
AvCystatin treatment reduced RSV-induced immunopathology in Th2-based models of viral lung eosinophilia. A) Schematic of the vvG RSV model: e.d. epidermabrasive; i.p. intraperitoneal; i.n. intranasal application. B) Weight loss in vvG RSV over an eight day period C) Eosinophil, neutrophil, and macrophage influx into BAL: total numbers in vvG RSV. D) RSV-specific serum immunoglobulin levels. E) L gene copy numbers in lungs, d4 after RSV/mock challenge. Naïve (light grey bars): mock infected and mock treated with PBS; vvG/RSV (black bars): scarified with vvG and challenged with RSV; vvG/AvCystatin/RSV (white bars): vvG/RSV plus intraperitoneal (d -14 and -7) and intranasal (d -2 and -1) injection of AvCystatin. Representative data from 2 independent experiments, 5 mice per group. Error bars indicate SEM. *P* values reflect Mann-Whitney t-test: * p<0.05, **p<0.01.

To analyse efficacy and safety of AvCystatin in virus-enhanced lung inflammation we first compared its effect in a model reflecting RSV-induced bronchiolitis (Fig 1A). The vvG/RSV model uses live attenuated vaccinia-virus as a vehicle for RSV-G protein instead of denaturation and adjuvant (day -14), and live virus challenge (d0). Pathology in vvG/RSV mice causes enhanced weight loss after RSV challenge (Fig 1B) accompanied by pulmonary inflammation with both eosinophils and neutrophils (Fig 1C). AvCystatin treatment [18] was administered during both the sensitization and challenge phases to the relevant sites (i.p. and i.n.) and time points (d-14, d-7 and d-2, d-1) (Fig 1A). This combined treatment significantly reduced vaccine-enhanced RSV immunopathology, reducing weight loss (Fig 1B), neutrophil and eosinophil influx (Fig 1C)), and mucus production in the airways (S4A Fig). However, levels of RSV-specific Immunoglobulin (Ig) G1 and IgG2a, measured 8 days post RSV infection, were unchanged (Fig 1D). Furthermore, CD8^+^ T-cell responses, including IFNγ production, were not affected by AvCystatin treatment (data not shown). Clinical immunosuppressants and some live helminth infections induce generalised suppression of immune responses and might thus result in enhanced virus load or extended virus persistence. However, AvCystatin did not increase the load or prolong the persistence of RSV in the lung indicating that beneficial antiviral immune responses were not suppressed (Fig 1E).

To assess the ability of AvCystatin to suppress enhanced Th2 pathology in an alternative model, we induced Th2-based disease by vaccinating mice with FI-RSV (S1A Fig). This scenario mimics the human Lot100 trails in the early 1960’s that enhanced disease severity in infants. In this case, treatment with AvCystatin completely prevented weight loss (S1B Fig). FI-RSV-driven eosinophil influx into the airways was also significantly reduced (S1C Fig) whilst macrophage numbers were enhanced (S1D Fig). Again we observed no increased viral load or prolonged persistence of RSV (S1E Fig). These findings again demonstrate that AvCystatin effectively reduces RSV-enhanced Th2-immunopathology without impairing antiviral responses.

### AvCystatin reduced chemokine and cytokine release in the airways

**Fig 2:**
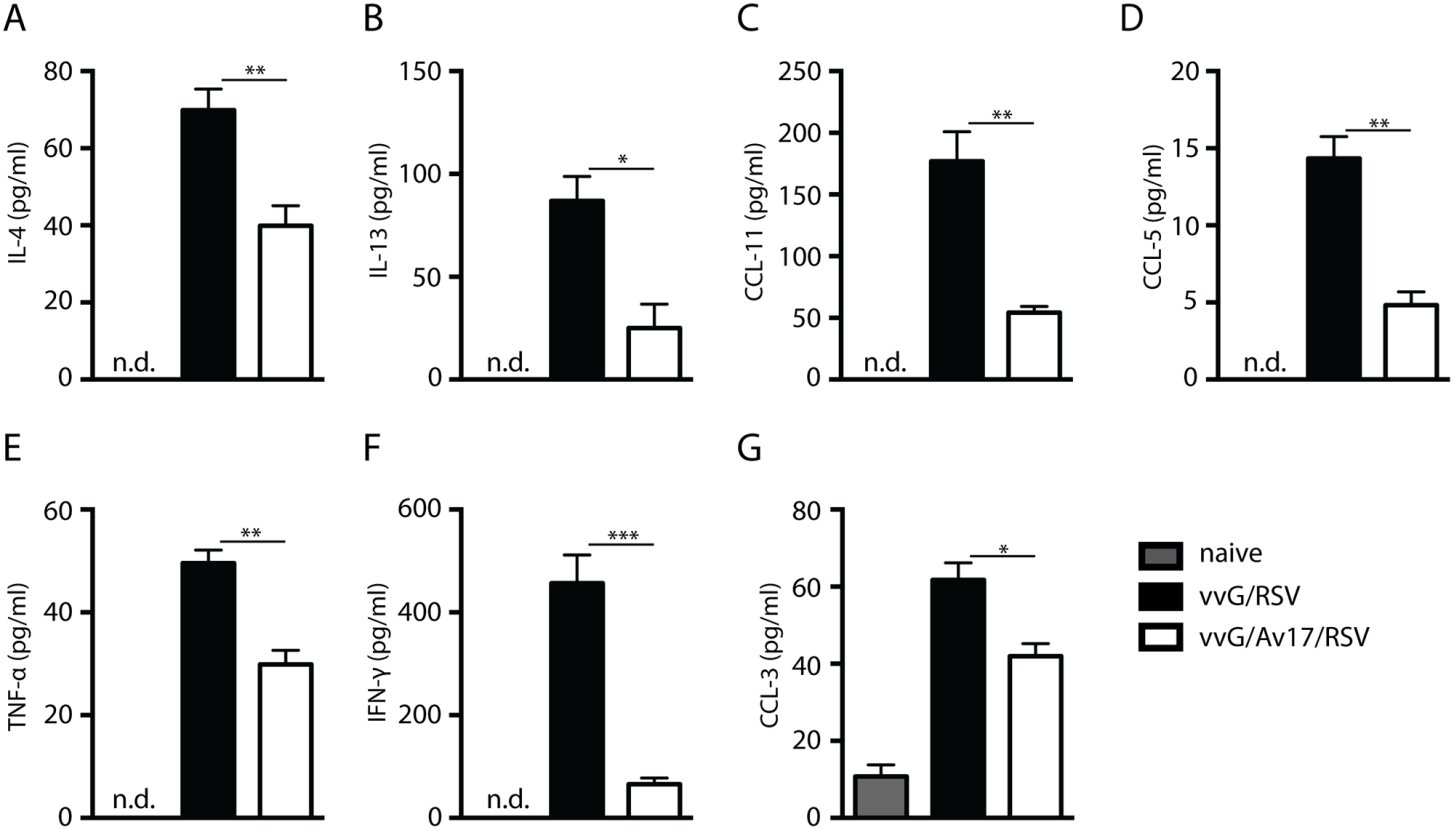
AvCystatin reduced chemokine and cytokine release in the airways. A) IL-4, B) IL-13, C) CCL11/Eotaxin, D) CCL5 / RANTES, E) TNF- α, F) IFN-γ, G) CCL3 / MIP-1α, Detection limit: 0.3pg/ml; 7.8pg/ml; 2.2pg/ml; 5.2pg/ml; 3.4pg/ml; 1.8pg/ml; 12.5pg/ml, respectively). Day 4 post RSV/mock challenge. Naïve (light grey bars): mock infected and mock treated with PBS; vvG/RSV (black bars): scarified with vvG and challenged with RSV; FI-RSV/AvCystatin/RSV (white bars). Representative data from 2 experiments, 5 mice per group. Error bars indicate SEM. *P* values reflect Mann-Whitney t-test: * p<0.05, **p<0.01, ***p<0.001.

Since cytokine expression in the airways reflects and influences the outcome of enhanced RSV disease, we analysed Bronchiolar Alveolar Lavage (BAL) fluid in the vvG/RSV model for the presence of both Th1 or Th2 specific cytokines and chemokines. Treatment of mice sensitised as described previously (Fig 1A) with AvCystatin significantly reduced the levels of IL-4, IL-13, eotaxin (CCL11) and CCL5/RANTES (Fig 2A–2D). TNF-α, IFN-γ and MIP1α (CCL3) were also suppressed (Fig 2E–2G), whereas IL-6 and IL-17 remained unaltered (data not shown). Thus, AvCystatin down-regulates the expression of specific Th1 and Th2 cytokines as well as chemokines implicated in RSV-induced airway pathology and eosinophilia, but does not cause general cytokine and chemokine suppression or interfere with antiviral defence.

### AvCystatin induces IL-10 production by CD4^+^ T cells in airways and lungs

**Fig 3:**
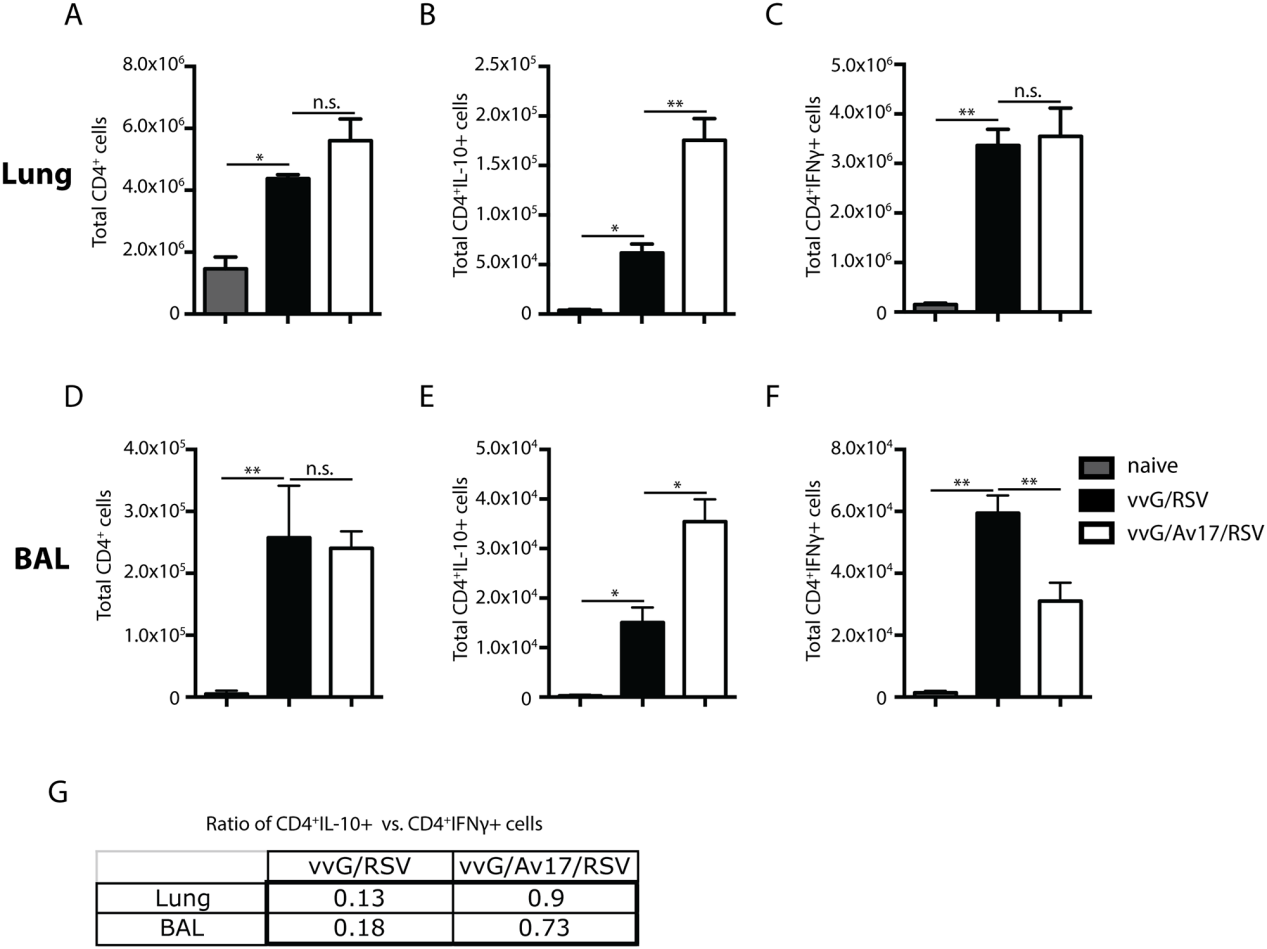
AvCystatin induced IL-10 production by CD4^+^ T cells in airways and lungs. Total CD4^+^ T cell counts in lungs (A) and BAL (B). CD4^+^ T cells were stimulated with PMA/ionomycin for 3h, IL-10^+^ and IFN-γ^+^ cells were gated in lung (B+C) and BAL (E+F). Ratios of IL-10^+^ versus IFN-γ^+^ CD4^+^ T cells in lung and BAL (G). Naïve (light grey): mock infected and mock treated with PBS; vvG/RSV (black bars): scarified with vvG (d-14) and challenged with RSV (d0); vvG/Av17/RSV (white bars): model plus injection of AvCystatin (d-14 and -7 i.p., d-2 and -1 i.n.). (A) Combined data from 2 independent experiments, (B-G) Representative data from 2 independent experiments, 5 mice per group. Error bars indicate SEM. *P* values reflect Mann-Whitney t-test: * p<0.05, **p<0.01.

To investigate the mechanisms responsible for the observed protection, we analysed cellular recruitment into airways, lungs and draining lymph nodes. Administration of AvCystatin induced an increase in the numbers of CD4^+^ T cells in the lungs at day 8 post RSV challenge (Fig 3A), but not in the airways (Fig 3D). Notably, both the number and frequency of CD4^+^ T cells producing IL-10 was increased in both lungs and airways (Fig 3B, 3E and S2A–S2E Fig) whereas the number of those producing IFN-γ was decreased in BAL (Fig 3F) but not in the lungs (Fig 3C). Notably, comparison of CD4^+^IL-10^+^IFN-γ^-^ with CD4^+^IL-10^-^IFN-γ^+^ populations showed a shift from pro-inflammatory to immunosuppressive environment (Fig 3G). Thus, the observed protective effects of AvCystatin were associated with the induction of IL-10 production by CD4^+^ T cells.

### AvCystatin reduces airway inflammation in primary (Th1) RSV infection

**Fig 4:**
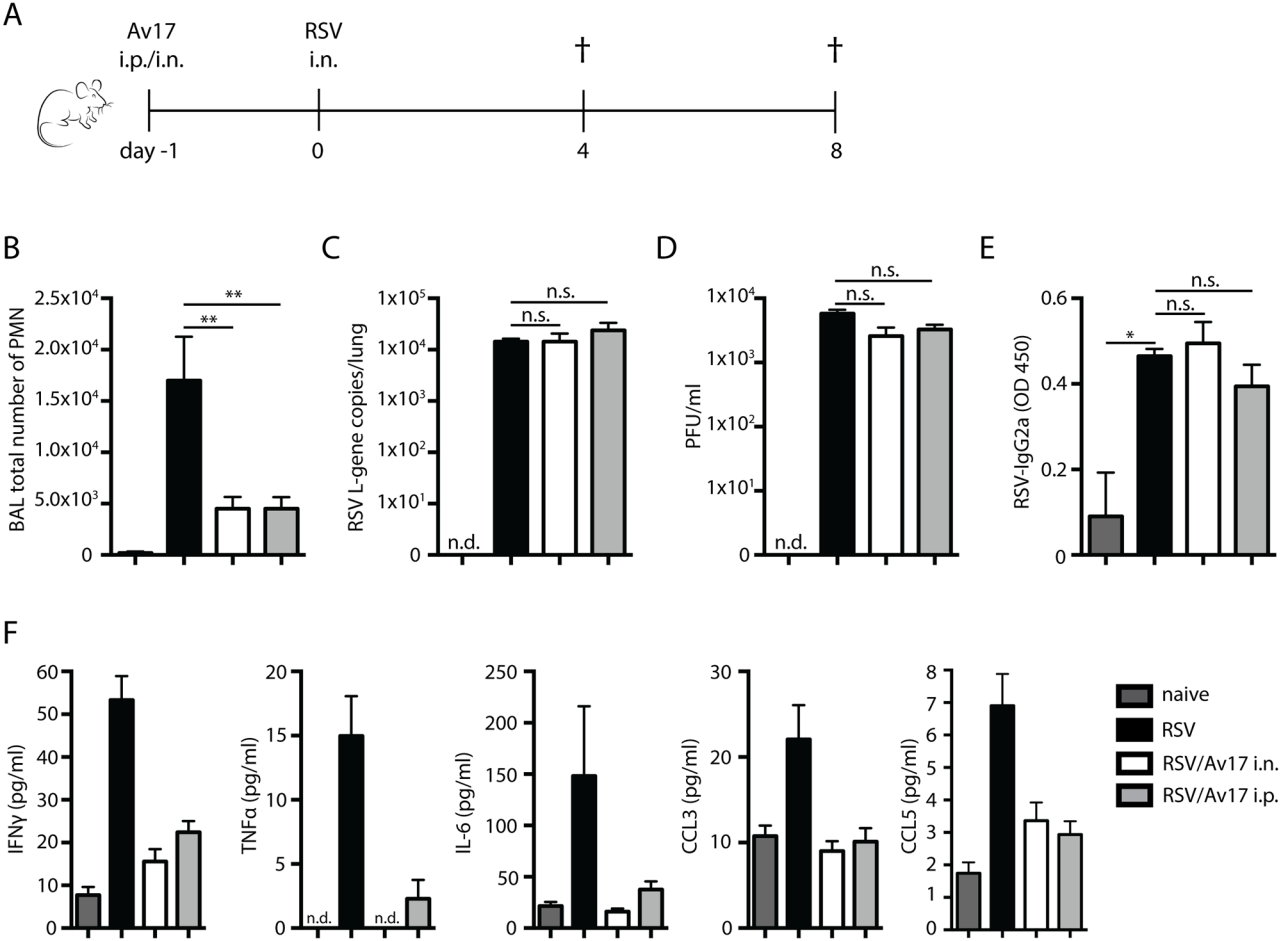
AvCystatin treatment in primary RSV infection. A) Schematic of the primary RSV model with AvCystatin treatment regimen: i.p. intraperitoneal; i.n. intranasal application. Neutrophil influx in the BAL was shown (B). RSV L gene copy numbers in the lungs (C) and viral load (D) measured 4 days post RSV/mock challenge. RSV-specific IgG2a detected in serum 8 days post infection (E). IFN-γ, TNFα, IL-6, CCL3, and CCL5 cytokine and chemokine production (F). Naïve (dark grey bars): mock infected and mock treated with PBS, RSV challenged day 0 (black bars); AvCystatin/RSV; AvCystatin treatment i.n. or i.p. on day -1 (white bars or light grey bars, respectively). Representative data of at least 2 independent experiments, 5 mice per group. Error bars indicate SEM. *P* values reflect Mann-Whitney t-test: * p<0.05, **p<0.01.

**Fig 5:**
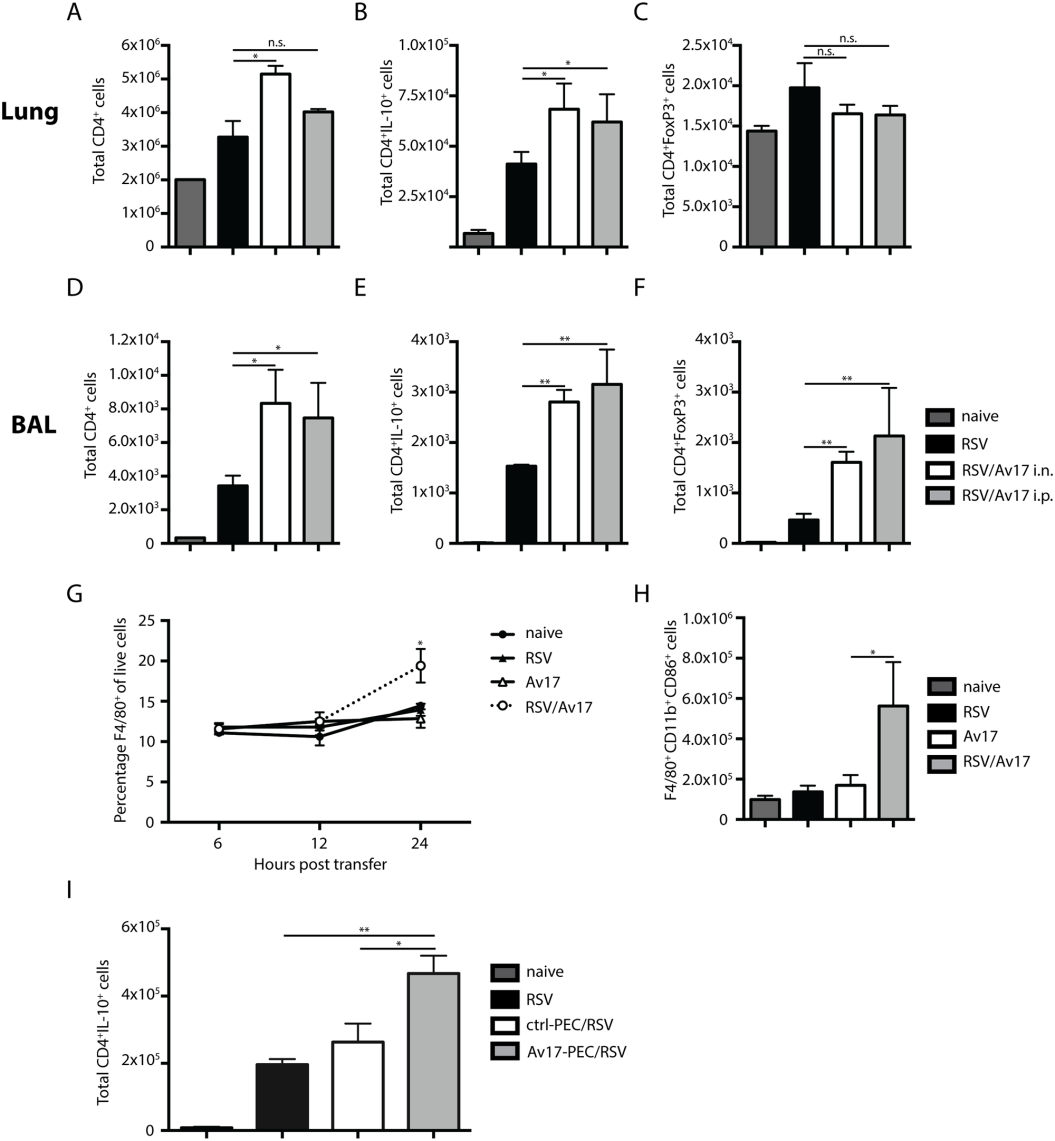
IL-10^+^ CD4^+^ T cell induction by AvCystatin conditioned macrophages. Lung and BAL CD4^+^ T cells detected by flow cytometry (A and D). Total number of IL-10 producing T cells determined by intracellular staining for IL-10 after restimulation with PMA/ionomycin for 3h in Lung and BAL (B and E). Number of CD4^+^ T cells positive for FoxP3 in lung (C) and BAL (F). Percentage of F4/80^+^CD11b^+^ cells infiltrating the lungs 24h post RSV challenge (G) and total cell number (H). Total cell number of CD4^+^ IL-10^+^ T cells infiltrating the BAL, 8 days post PEC transfer (I). Naïve (dark grey bars): mock infected and mock treated with PBS, RSV challenged day 0 (black bars); AvCystatin/RSV; AvCystatin treatment i.n. or i.p. on day -1 (white bars or light grey bars, respectively). Representative data of 2 experiments, 5 mice per group. Error bars indicate SEM. *P* values reflect Mann-Whitney t-test: * p<0.05, **p<0.01.

We next investigated whether the protective effects of AvCystatin were limited to Th2-induced pathologies or depended on the route of application. Primary RSV infection causes a predominantly Th1 response, with an influx of neutrophils to the airways by day 4, and T cell-dependent pulmonary immunopathology by day 8 post infection (Fig 4A). Either intranasal (i.n.) or intraperitoneal (i.p.) administration of AvCystatin significantly reduced influx of neutrophils to the airways by day 4 (Fig 4B) without affecting viral load and CD8^+^ T-cell responses (Fig 4C and 4D, and data not shown). Furthermore, intranasal administration significantly reduced MUC5a expression in the lungs (S4B Fig). AvCystatin treatment did not alter serum IgG2a levels measured 8 days post infection (Fig 4E). However, administration of AvCystatin via either route suppressed expression of IFN-γ, TNF-α, IL-6, CCL3/MIP-1α and CCL5/RANTES in the airways (Fig 4F). Mirroring the vaccine enhanced vvG/RSV model (Fig 1A), we observed an increased influx of CD4^+^ T cells in the lungs and airways in AvCystatin treated mice undergoing primary infection (Figs 4A, 5A and 5D). Moreover, the infiltrating CD4^+^ T cells revealed an increased frequency of IL-10 producers after either i.n. or i.p. administration of AvCystatin (Fig 5B and 5E) accompanied by an remarkable increase in CD4^+^CD25^+^Foxp3^+^ positive Treg cells in the airways (Fig 5F), but not in the lungs (Fig 5C). These results indicate that AvCystatin also selectively protect mice from excessive inflammation during primary RSV infection.

Additionally, examination of cells from the regional nodes showed that intraperitoneal, but not intranasal, AvCystatin induced systemic CD4+FoxP3+ cells; in contrast, only intranasal treatment induced LN IL-10 production (S3A and S3B Fig). Thus, AvCystatin induced regulatory T cells and especially CD4^+^IL-10^+^ T cells in primary RSV infection, limiting RSV induced immunopathology.

### AvCystatin conditioned macrophages induce IL-10 production in airway CD4^+^ T cells

Previous studies [18,20], indicate that macrophages may be involved in causing the immunoprotection induced by AvCystatin. We therefore looked at the role and function of these cells in primary RSV infection. We found that recruitment of F4/80^+^CD11b^+^ macrophages was enhanced by AvCystatin treatment *via* airways 24h after RSV challenge (Fig 5G and 5H). Interestingly, lung F4/80^+^ macrophages showed no increase in production of IL-10 at 4 and 8 days post-RSV infection (data not shown) although peritoneal macrophages have been shown to bind AvCystatin [20] and migrate to the spleen and mediastinal lymph nodes after adoptive transfer [25]. To determine whether AvCystatin primed macrophages might induce IL-10 production by CD4^+^ T cells, we primed peritoneal exudate cells (PEC; 70% macrophages and 30% B cells) with AvCystatin and adoptively transferred these cells to naïve mice prior to RSV infection. Transfer of AvCystatin-primed PEC resulted in elevated numbers of IL-10^+^ CD4 T cells in the airways at 8 days post-RSV infection (Fig 5I). In contrast, PECs primed with heat-inactivated AvCystatin prior to transfer had no effect. PECs therefore appear to be crucial in the uptake and processing of active AvCystatin, leading to the induction of IL-10-producing CD4^+^ T cells which may mediate protective effects observed in the lung. This observation complements previous research showing protection by AvCystatin treated macrophages, via the induction of IL-10 producing CD4^+^ T-cells, in an experimental model of DSS colitis [25].

## Discussion

Testing the therapeutic potential of AvCystatin in viral lung disease, we show that it has beneficial effects in both primary and in vaccine-enhanced lung pathology. It reduces immune-mediated disease that is seen in RSV infection in its various forms without impairing antiviral defences. It beneficial effects appear to be mediated via macrophages that promote regulatory and IL-10 producing CD4^+^ T cells, and include a potent inhibition of eosinophilic lung disease.

These findings help to explain epidemiological findings that show an inverse relationship between the prevalence of parasitic worm infection and that of allergy and autoimmunity [26–28]. This correlation is often explained in the context of the hygiene hypothesis, which suggests that diseases characterized by an over-reactive immune response against harmless or self-antigens are caused by a lack of immunological ‘training’, related to a reduced rate of infections that were once common but are now mainly found in developing countries [29], [30].

Epigenetic ‘imprinting’ has been recently implied by the observation of protective effects of previous bacterial infections on RSV disease [31,32]. An effect of parasite colonisation on RSV disease would seem to be via different mechanisms, since Th2-driven immunopathology (as observed in allergic inflammation), shares many of the features of anti-helminth parasite immune responses (e.g. eosinophilia, production of IgE, IL-13 and eotaxin). Parasites appear to have evolved complex mechanisms whereby they inhibit Th2 immune responses, presumably to survive better within their hosts. There is some evidence that these immunomodulatory mechanisms may also suppress unrelated allergic and inflammatory responses, but the relationships and mechanisms are complex and difficult to investigate in man. However, experiments in mice show that helminth infections can abrogate allergic airway inflammation [11],[12] and co-infection studies indicate that helminths may enhance susceptibility to viruses. In *Schistosoma mansoni* and lymphocytic choriomeningitis virus (LCMV) infection in mice, helminth-related immunosuppression of antiviral type I IFN responses resulted in infiltration of high numbers of LCMV-specific CD8 cells into the liver, leading to increased morbidity and hepatotoxicity [16]. Additionally, latent herpes infection could also be reactivated by helminthic worms in a STAT6 dependent manner, by the production of IL-4 [33]. Or in the case of Norovirus by inhibition of T cell proliferation by alternatively activated macrophages developing in the presence of IL-4 [34]. The use of purified recombinant immunomodulators rather than live helminth infection provides a controlled means by which to alleviate immunopathology [9].

In our present studies, AvCystatin suppressed Th2 cytokine production (especially IL-4), minimising the recruitment of inflammatory cells into the airways. Furthermore, production of chemokines linked to eosinophilia (RANTES and eotaxin) was strongly inhibited, and this was reflected by the inhibition of virus-related eosinophilia. However, AvCystatin did not generally suppress cytokine production, allowing for effective antiviral responses, as for example IL-6 expression was unaltered. IL-6 is considered a pro-inflammatory cytokine induced in RSV infection [35] and known to be crucial for follicular helper cell and germinal centre development in chronic virus infection [36] and acute antiviral responses [37]. AvCystatin thus modulates rather than generally suppresses immune responses, a feature also seen in some long persisting helminths (e.g. *Heligmosomoides polygyrus* [11]).

Recent work confirms a crucial role for IL-10 in the outcome of RSV infection, with T cells identified as the major source [38–40]. In the current study, we observed induced numbers of F4/80+CD11b+ macrophages, identified as the major population of cells in peritoneal exudates which ingest AvCystatin [20]. Previous *in vitro* and *in vivo* studies (OVA airway hyperreactivity), indicate a key role for IL-10 and macrophages in the AvCystatin-induced amelioration of inflammation [18,20]. Application of AvCystatin in vvG- RSV and primary RSV infection models resulted in the induction of IL-10-producing CD4^+^ T cell populations. By adoptive transfer of AvCystatin-primed peritoneal exudate cells we were able to induce IL-10 producing CD4 T cells and ameliorate airway inflammation. This finding is in support of the demonstration that adoptive transfer of AvCystatin-primed macrophages, but not B cells, mediated protection in a model of allergic airway inflammation and colitis via induction of IL-10 producing T cells [25]. The current study shows that exposure to AvCystatin leads to enhanced recruitment of IL-10-producing CD4^+^ T cells in viral infection; however, FoxP3 expression did not coincide with IL-10 production in the regional lymph nodes. The source of IL-10 can therefore differ depending on the infection, tissue environment and type of inflammatory insult. However, we do not rule out a role for FoxP3+ Treg cells in the control of RSV pathology, as we have observed an increase in Treg cells in the airways after AvCystatin treatment during primary infection and previous work has implicated a role for Treg cells in suppressing RSV immunopathology [2], [41], [42].

In relation to human RSV-induced bronchiolitis, neutrophils appear to be the instigators of pathology and dampening neutrophillic inflammation by AvCystatin or other neutrophil suppressors, may well be a way forward for future patient care. However, due to the proposed antiviral function of neutrophils, current practice suggests to target neutrophils under the umbrella of antiviral treatment [43].

This is, to our knowledge, the first time that a recombinant parasite-derived immunomodulator has been shown to down-regulate virally-enhanced inflammation without impairing responses important for anti-viral immunity. We provide evidence that AvCystatin immunomodulation may act through the production of CD4^+^ T cell derived IL-10. Collectively, our findings support the testing of AvCystatin as a potential novel agent to counter virus-enhanced respiratory inflammation.

## Materials and Methods

### Ethics statement

All mouse experiments were ethically approved by the Imperial College Central Biological Services (CBS) ethics committee performed in accordance with approved UK Home Office guidelines (Project License No. PPL 70/6785).

### AvCystatin

Mice were treated with 20 μg AvCystatin prepared as previously described [18] or PBS intraperitoneally (i.p.) or intranasally (i.n.).

### Viral infection

RSV (strain A2) and rVV-G (vaccinia virus expressing the RSV G-protein) were propagated in Hep-2 cells (ATCC) as described previously [44]. FI RSV was obtained by formalin inactivation of RSV A2 according to FI RSV Lot 100 protocols, with alum as adjuvant [45]. For FI-RSV models, eight week old female BALB/c mice (Harlan, UK) were injected with FI-RSV intramuscularly (day -14) and infected intranasally with 5 x 10^5^ PFU RSV 2 weeks thereafter. For rvv-G RSV models, mice were primed with vaccinia expressing RSV-G protein (3 x 10^6^ PFU) by scarification of the rump and infected intranasally with 5 x 10^5^ PFU RSV 14 days later. RSV titres were assessed by titration of lung homogenates on Hep-2 cell monolayers [46]. RSV L-gene copy numbers were determined by TaqMan technology using the delta delta ct method, and normalization with 18S as previously described [47].

### Bronchoalveolar lavage (BAL) and cell isolation

BAL-accessible cells were obtained by repeated instillation of 1 ml of PBS/12 mM lidocaine via the trachea in sacrificed animals. Cytospin preparations were stained with hematoxylin and eosin and analysed by light microscopy (300 cells per slide). Lungs were homogenized and digested with collagenase D (50ug/ml, Sigma, Gillingham, UK) for 30 min. Mediastinal (peribronchial) lymph nodes were dissociated and filtered through a 100 μm cell strainer.

### Flow cytometry

Flow cytometry was performed as previously described [38]. Briefly, cells were incubated with LIVE/DEAD Fixable cell stain (Invitrogen), then stained for 20 min with: MHCII-PacificBlue, F4/80-APC (Biolegend), CD4-AF700, CD8-PacificBlue, CD25-APC, CD86-APC, CD49b-Pe-Cy7 (eBioscience), CD11b-PE-Cy7, CD45R/B220-PerCP-Cy5.5 (BD-Pharmingen). For intracellular staining, the samples were stimulated for 3 h (100 ng/ml phorbol 12-myristate 13-acetate, 1 μg/ml ionomycin; monensin added after 1 h). Cells were fixed and permeabilized with Cytofix/Cytoperm (BD), then stained with IL-10-FITC, IFN-γ-PerCP-Cy5.5, IL-17-PE, FoxP3-AF488 (BD-Pharmingen) antibodies before analysis on LSRII flow cytometer (BD), collecting 100,000 live events. Data was analyzed using FlowJo (v7.6.5, Tree Star, Ashland, OR, USA).

### Adoptive transfer of peritoneal exudate cells (PEC)

Donor BALB/c were injected i.p. with 20 μg active or heat-inactivated AvCystatin and peritoneal exudate cells (PEC) recovered 20 h later in PBS/2 mM EDTA/0.2% BSA. Recipient mice were injected intravenously with 3x10^6^ PEC and infected with RSV i.n. 4 h later.

### Cytokine and Chemokine detection

Quantification of BAL chemokines and cytokines was performed using a 13-plex Luminex kit (Millipore, Watford, UK). Data were acquired with a Luminex 100 (Applied Cytometry systems, Sheffield, UK) and Starstation software.

### Statistical analysis

GraphPad Prism software (La Jolla, CA, USA) was used to analyse data, generally shown as mean ±SEM of 5 animals per group. Mann-Whitney t-test and 2 way ANOVA were used to compare data. *P* values of <0.05 were considered significant.

## Supporting Information

S1 Fig Reduced RSV-induced immunopathology by AvCystatin treatment in a model of FI-RSV of viral lung eosinophilia. A) Schematic of the FI RSV model: i.m. intramuscular; i.p. intraperitoneal; i.n. intranasal application. B) Weight loss in the FI-RSV model. Total cell number of eosinophil (C) and macrophages in the BAL (D). Viral load in the lungs measured by RSV L-gene copies (E). Representative data of 2 experiments, 5 mice per group. Error bars indicate SEM. *P* values reflect Mann-Whitney t-test: * p<0.05, **p<0.01.
(TIF)

S2 Fig Frequency of IL-10 producing T-cells in the lungs and airways after AvCystatin treatment. Flowcytometric analysis of IL-10 intracellular cytokine content in RSV challenged or AvCystatin/RSV challenged mice is shown (A). Graphical visualization of IFNγ and IL-10 production by CD4^+^ T cells in the BAL (D and E) and lungs (B and C). Representative data of 2 experiments, 5 mice per group. Error bars indicate SEM. *P* values reflect Mann-Whitney t-test: * p<0.05, **p<0.01.
(TIF)

S3 Fig AvCystatin induced FoxP3^+^ T cell induction in the mediastinal lymph node. Total number of FoxP3^+^ CD4^+^ T cells (A) and the number of IL-10^+^ CD4^+^ T cells in the mLN (B) after AvCystatin treatment and RSV challenge. Representative data of 2 experiments, 5 mice per group. Error bars indicate SEM. *P* values reflect Mann-Whitney t-test: * p<0.05, **p<0.01.
(TIF)

S4 Fig AvCystatin treatment reduces Muc5a production in lung. Relative expression of MUC5a in mice lungs after the vvG RSV model (A) or primary RSV model (B). Error bars indicate SEM. *P* values reflect Mann-Whitney t-test: * p<0.05.
(TIF)
