# Supplementary figures and images for: The Helminth-Derived Immunomodulator AvCystatin Reduces Virus Enhanced Inflammation by Induction of Regulatory IL-10+ T Cells
Source: PLoS One. 2016 Aug 25;11(8):e0161885. doi: 10.1371/journal.pone.0161885 (PMC4999285; doi:10.1371/journal.pone.0161885)

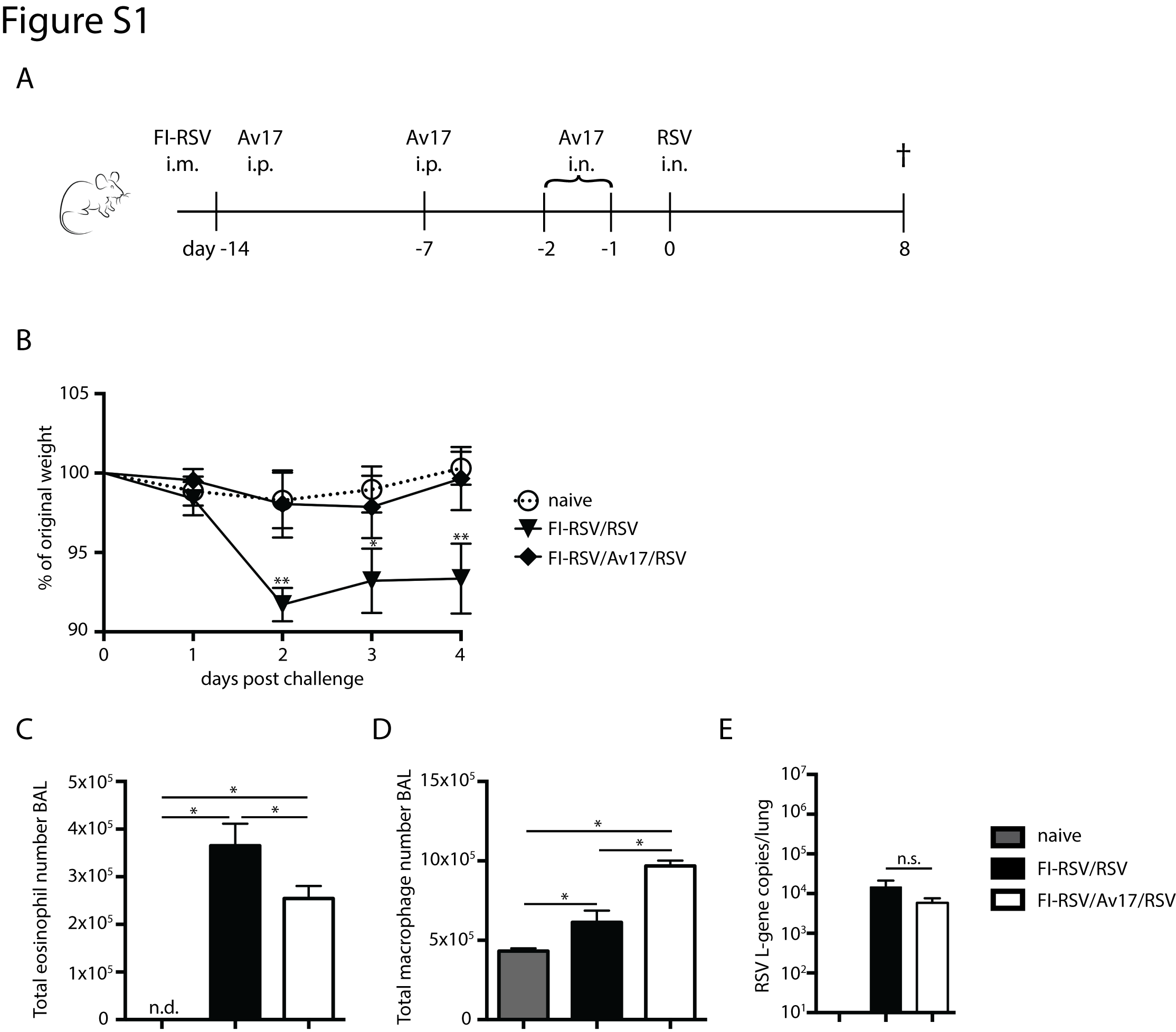

Supplement: S1 Fig — A) Schematic of the FI RSV model: i.m. intramuscular; i.p. intraperitoneal; i.n. intranasal application. B) Weight loss in the FI-RSV model. Total cell number of eosinophil (C) and macrophages in the BAL (D). Viral load in the lungs measured by RSV L-gene copies (E). Representative data of 2 experiments, 5 mice per group. Error bars indicate SEM. P values reflect Mann-Whitney t-test: * p<0.05, **p<0.01. (TIF) [file pone.0161885.s001.tif]

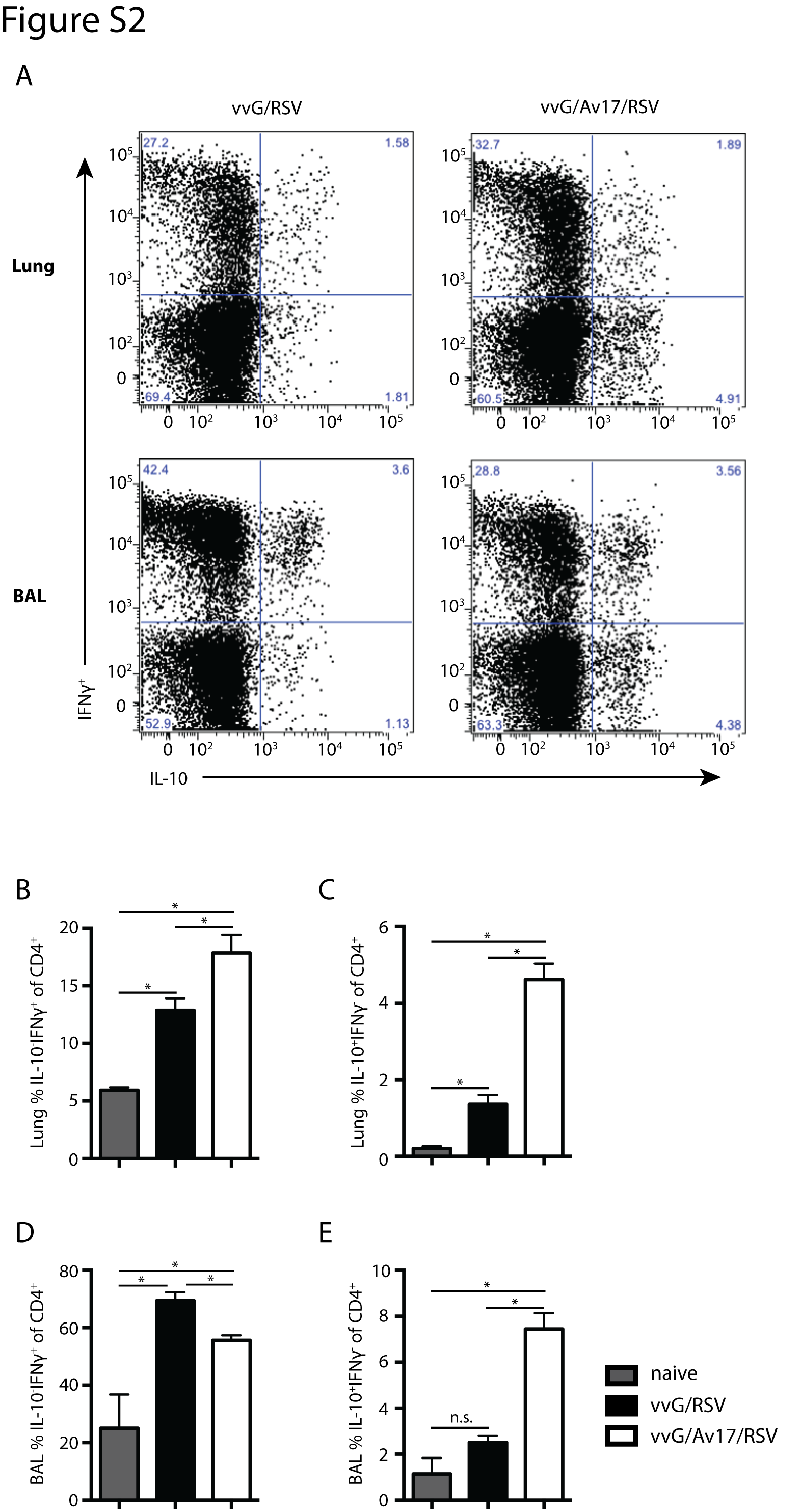

Supplement: S2 Fig — Flowcytometric analysis of IL-10 intracellular cytokine content in RSV challenged or AvCystatin/RSV challenged mice is shown (A). Graphical visualization of IFNγ and IL-10 production by CD4+ T cells in the BAL (D and E) and lungs (B and C). Representative data of 2 experiments, 5 mice per group. Error bars indicate SEM. P values reflect Mann-Whitney t-test: * p<0.05, **p<0.01. (TIF) [file pone.0161885.s002.tif]

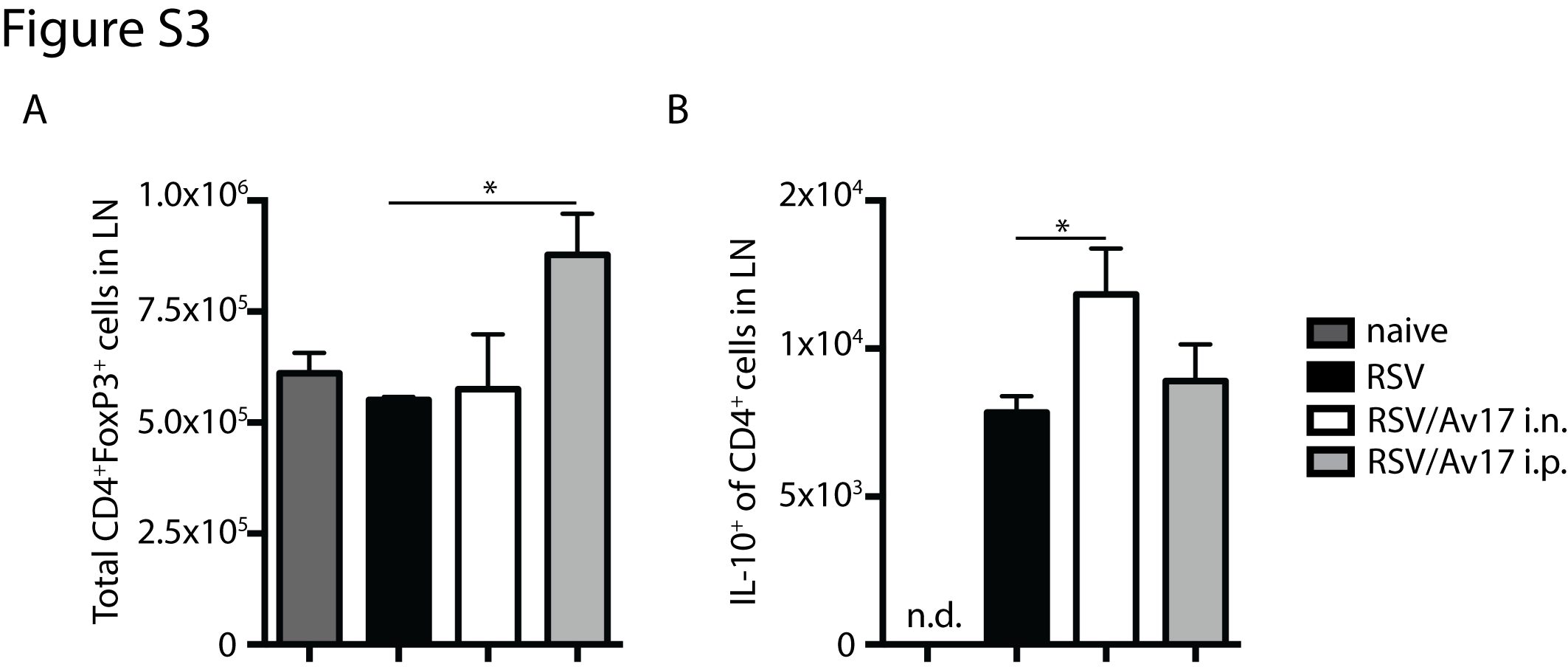

Supplement: S3 Fig — Total number of FoxP3+ CD4+ T cells (A) and the number of IL-10+ CD4+ T cells in the mLN (B) after AvCystatin treatment and RSV challenge. Representative data of 2 experiments, 5 mice per group. Error bars indicate SEM. P values reflect Mann-Whitney t-test: * p<0.05, **p<0.01. (TIF) [file pone.0161885.s003.tif]

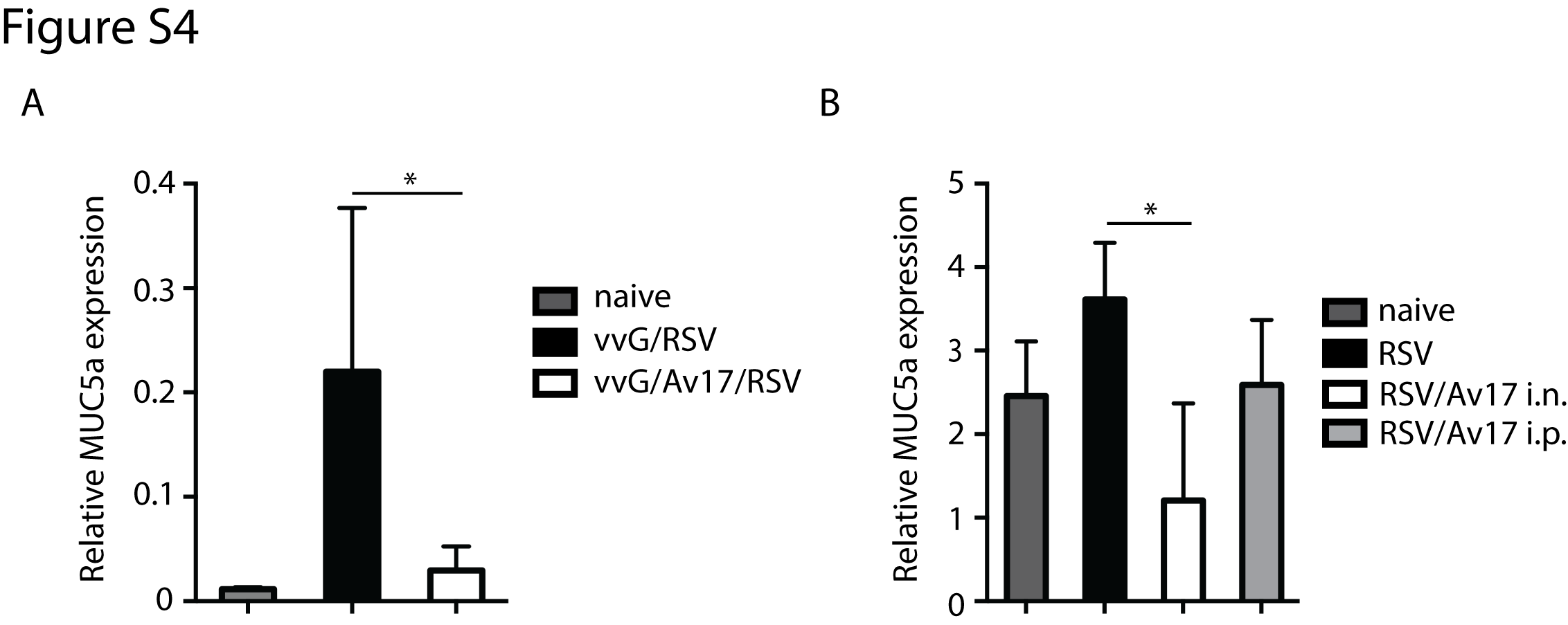

Supplement: S4 Fig — Relative expression of MUC5a in mice lungs after the vvG RSV model (A) or primary RSV model (B). Error bars indicate SEM. P values reflect Mann-Whitney t-test: * p<0.05. (TIF) [file pone.0161885.s004.tif]
